# Mutual information-based hierarchical NBV decision for active semantic visual SLAM under dynamic environments

**DOI:** 10.1038/s41598-026-36259-x

**Published:** 2026-01-20

**Authors:** Zhenyuan Yang, Ash Wan Yaw Sang, M. A. Viraj J. Muthugala, Mohan Rajesh Elara

**Affiliations:** Engineering Product Development, Singapore University of Technology and Design, Singapore, 487372 Singapore

**Keywords:** Engineering, Mathematics and computing

## Abstract

Active Simultaneous Localization and Mapping (A-SLAM) technology enables a robot to autonomously plan its movements to build a comprehensive and accurate map of its surroundings. However, most existing SLAM algorithms are not robust in dynamic environments, as moving objects can negatively impact mapping and localization accuracy, making it difficult for the robot to keep tracking and fully understand its environment. While some semantic SLAM methods can identify and exclude dynamic objects, in active SLAM, excluding features without proper path planning carries significant risks of losing track. In this work, we propose a real-time mutual information-based active SLAM approach designed to enhance robustness in dynamic environments. The proposed method not only excludes dynamic objects from the mapping process but also integrates two Next-Best-View (NBV) decision modules to improve path planning and maintain robustness. This feature allows for improved mapping efficiency and robustness to avoid losing tracking in dynamic environments. Experiments conducted in two simulated environments and one real-world scenario demonstrate that the proposed active SLAM algorithm maintains its robustness and efficiency in dynamic environments, and is deployable in real applications.

## Introduction

Simultaneous Localization and Mapping (SLAM) has long been a critical area of focus in robotics, particularly in autonomous navigation and path planning. Visual-based SLAM is more widely used because of its low cost and higher sensitivity in the textures and color in the environments^1^ compared to laser-based SLAM. Traditional visual SLAM methods such as ORB-SLAM2^2^, LSD-SLAM^3^, and VINS^4^ all have good performance across typical scenes. Active SLAM means a robot autonomously creating a map of its environment, localizing itself, and controlling its own movements^5^. Unlike passive SLAM, active SLAM does not rely on manual intervention or predefined waypoints, making it more adaptable to unpredictable and unfamiliar real-world environments.

In real-world scenarios, dynamic elements such as moving people are common and must be considered. These dynamic objects can significantly impact the accuracy of a robot’s localization. This challenge is further intensified by the inherently limited Field Of View (FOV) of cameras in visual SLAM, which prevents the robot from capturing a sufficiently rich set of static features in a single observation and makes the system more vulnerable to occlusion by moving objects. To address this challenge, semantic SLAM techniques, such as DS-SLAM^6^ and Dyna-SLAM^7^, have been developed for dynamic environments. These methods use semantic segmentation to exclude dynamic objects by excluding all the dynamic features.

In passive visual SLAM, tracking can be corrected through manual control by moving the robot to areas with rich features. In active SLAM, however, the robot must plan its own movements. While many active visual SLAM methods focus on efficient or complete mapping^8,9^ , robustness and stability are equally important to avoid tracking loss, especially in dynamic environments where dynamic features are excluded. Traditional path planning algorithms often overlook moving obstacles and their relative motion with the robot. They typically maximize information gain on an occupancy map without modeling the predicted spatiotemporal occupancy of moving objects or their relative motion with the robot. As a result, paths that pass close to a pedestrian can continually reduce visible static features after semantic masking, leading to the exclusion of a large portion of the features in the current frame, increasing the risk of feature tracking failure. Similarly, if the robot continues to move in the same direction as the object, fewer features will be consistently captured, resulting in reduced mapping and localization accuracy. Therefore, proper motion planning is essential for active semantic SLAM in dynamic environments to ensure robust performance.

This paper proposes a novel hierarchical active semantic visual SLAM system based on the information theory^10^ to increase robustness under dynamic environments. The system has a module to find the global Next-Best-View (NBV) for the robot. Further, the system can generate a Feature Probability Map (FPM) based on the current image input and choose the local NBV. For global and local NBV decisions, they are both based on the Shannon mutual information^11^. The main contributions of the proposed paper are as follows:

- A global NBV module is proposed to apply mutual information on an occupancy grid, designed to deal with the limited FOV of a camera.
- A pixel-level dynamic object prediction mechanism that models tracked object motion with anisotropic Gaussian distributions and fuses it with feature distributions to generate an FPM.
- A local NBV module that applies the FPM to select viewpoints with maximal expected feature observability while minimizing disturbance from dynamic obstacles.
- Comprehensive validation in both simulation and real-world environments, demonstrating that the proposed semantic active SLAM framework improves robustness and localization reliability in dynamic environments.

## Related work

### Active SLAM and robot exploration

Active SLAM emphasizes viewpoint selection by actively exploring unknown areas to improve mapping quality and localization robustness. Generally, exploration methodologies can be classified into learning-based and classical models, with classical models further divided into deterministic and stochastic approaches. Among deterministic approaches, the frontier-based method proposed by Yamauchi et al.^12^ is the most commonly used due to its simplicity and efficiency, but it lacks a measure of information gain in the environments. To address this, more advanced information-theoretic exploration methods were developed, which will be discussed detailedly in “Mutual information-based active SLAM”. Other deterministic strategies include Voronoi-based coverage planners^13^^14^, which applies a topological Voronoi graph instead of a heavy occupancy grid, making it easier to store, update, and plan long-range navigation. Stochastic models are advantageous when the exploration space is large or highly uncertain. Xu et al.^15^ developed an exploration planner combining incremental sampling with Probabilistic Roadmaps^16^, balancing efficiency and adaptability. Wang et al.^17^ introduced Semantic Road Maps, which detect frontiers and integrate both information gain and travel cost to improve efficiency in multi-room exploration. Bio-inspired neural approaches such as Glasius-based Neural Network^18^ generate smooth, adaptive trajectories by unifying obstacle avoidance, goal attraction, and exploration within a single neural field, offering robustness to noise and dynamic changes^19,20^. Recent work also has highlighted the importance of considering environment changes when exploration. Zhao et al.^21^ propose a predictive framework that combines recurrent temporal models with spatial representations to capture environment evolution over time. This paper opens new insights for active SLAM in dynamic environments, where exploration decisions can anticipate scene evolution rather than rely solely on instantaneous observations.

### Mutual information-based active SLAM

Mutual information has been widely used in SLAM-based exploration to optimize viewpoint selection by quantifying the information gain. It was developed and applied in maximizing map entropy reduction ?, or minimizing map variance ,. Recent research has explored various applications of mutual information in autonomous exploration and decision-making. For geometry information such as occupancy map, Michael et al.^22^ proposed a method to reduce communication overhead in multi-agent systems using Gaussian processes for spatial field estimation to let agents optimize the information-gathering process. Zhang et al.^23^ proposed a method for the efficient computation of Shannon mutual information to evaluate potential information gain from different sensing actions, thereby improving mapping efficiency. Except for geometry mutual information, semantic information can also be applied. Zhang et al.^24^ introduce an active metric-semantic SLAM approach that combines semantic mutual information with the connectivity metrics of the underlying pose graph to select a strategy during exploration. Asgharivaskasi et al.^25^ proposed a Bayesian multi-class mapping algorithm using an octree structure to compute Shannon mutual information efficiently, enabling autonomous robots to perform semantic exploration in unknown environments. Recent advances in temporal environment modeling ? suggest that explicitly predicting environment evolution can be critical when planning under uncertainty. These observations indicate a gap between classical information-theoretic exploration methods and the requirements of dynamic real-world environments.

### Semantic active SLAM

In active SLAM, semantic information has recently been increasingly applied to provide robots with a richer understanding of the environment during exploration, going beyond purely geometric methods. Fredriksson^26^ introduced a semantic topometric exploration strategy that segments the grid map into structural regions and exploits both metric and semantic frontier properties to achieve faster and more efficient exploration. Tao et al.^27^ proposed an active metric-semantic SLAM framework for aerial robots that balances exploration efficiency with localization uncertainty reduction. In addition to exploration, exploitation also plays a critical role in ensuring localization accuracy and robustness. Wasserman et al.^28^ presented exploitation-guided exploration, a modular navigation framework that integrates exploration and exploitation modules to improve semantic navigation accuracy and efficiency. Tian et al.^29^ developed a semantic-centered ground–air collaborative mapping and navigation framework that fuses UAV and UGV maps into a consistent global representation, improving both mapping precision and exploration efficiency.

While these approaches are effective in static environments, handling dynamic environments remains a significant challenge. In passive SLAM, semantic information has been widely used to improve robustness under dynamic conditions^6,7,30^. Recent semantic SLAM systems have increasingly focused on handling dynamic environments. Islam et al.^31^ integrated YOLO-based object detection with enhanced strategies of loop closure to improve long term robustness in dynamic scenes. Similarly, Islam et al.^32^ propose an adaptive segmentation framework combined with dynamic object detection, demonstrating improved performance in real-world environments with moving objects.

Several methods further couple semantic perception with geometric reasoning. ARD-SLAM^33^ introduces dynamic object identification alongside improved multi-view geometric constraints to achieve accurate and robust SLAM under scene dynamics. MVS-SLAM^34^ tightly integrates semantic RGB-D information with enhanced multi-view geometry, allowing dynamic regions to be identified while preserving static structure for mapping. FADM-SLAM^35^ emphasizes computational efficiency while maintaining robustness in environments with movable objects.

This work integrates mutual information-based NBV selection with semantic segmentation and dynamic object tracking. Unlike prior studies that mainly target static environments, our approach explicitly models moving objects as pixel-level Gaussian distributions and adapts both global and local NBV strategies to maintain robust localization and efficient exploration in dynamic environments.

## Mutual information-based NBV selection

### Framework overview

**Fig. 1:**
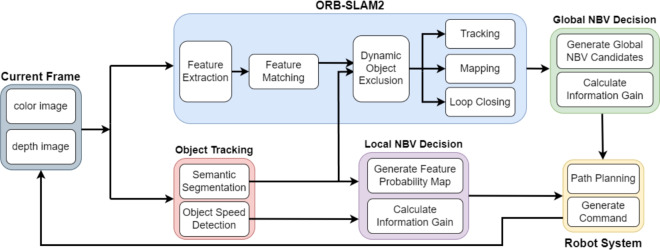
Overview of the proposed system. The system uses ORB-SLAM2 as the base. Object Tracking is done using Yolov8^36^ and BoT-SORT^37^. The two NBV modules that will be introduced in this work can let the robot do active exploration. The global NBV decision module will help the robot find a global goal based on map information gain. The local NBV decision module will let the robot choose which direction to go based on the information gain of two FPMs.

ORB-SLAM2^2^ is a widely used SLAM system with good performance, making it an excellent foundation for this work. In addition, this work focus on active SLAM with pure vision sensor without inertial measurements. When the IMU is disabled, the core visual frontend and backend optimization pipelines of ORB-SLAM3 are largely equivalent to those of ORB-SLAM2, except for additional components such as multi-map management and enhanced tracking recovery. ORB-SLAM2 therefore provides a simpler and more lightweight implementation that is sufficient for evaluating the proposed hierarchical NBV and semantic modules. The proposed system builds on ORB-SLAM2, integrating an active exploration module and enhancing it to adapt to dynamic environments. The overall framework of the proposed SLAM system is illustrated in Fig. 1. The robot’s decision variable is the selection of future viewpoints through motion actions. This work formulates two coupled optimization problems, one is global NBV problem that selects viewpoints to reduce geometric map uncertainty, the other is a local NBV problem that selects short-horizon motions to reduce feature observability uncertainty. These problems are solved hierarchically and are formalized in the following sections. The following subsections introduce the mutual information-based global exploration approach, the semantic segmentation and object tracking network, the process for detecting and excluding dynamic objects, and the function for local dynamic obstacle avoidance.

### Global NBV decision

For global exploration, the objective is to incrementally construct a complete 2D occupancy grid map, denoted as $G \subset \mathbb {R}^2$. During mapping, a 3D dense map is built by back-projecting depth images into point clouds, as described in^2^. The 2D occupancy grid is then generated using OctoMap^38^ by projecting point clouds within a specified height range onto a planar surface. Each grid cell $g \in G$ can take three semantic states: free, occupied, or unknown.

#### Probabilistic and entropy model

The environment is represented by a two-dimensional occupancy grid $G = \{ g_1, g_2, \dots , g_N \}$, where each grid cell $g_i$ is modeled as a Bernoulli random variable indicating whether the cell is occupied or free. The belief over each cell $p_t(g_i)$ at time *t* is defined in (1), where $z_{1:t}$ denotes the set of all observations up to time *t*. Cells labeled as free or unknown correspond to $p(g \mid z_{1:t})$ close to 0 or 0.5, respectively. The uncertainty of the occupancy grid is quantified using Shannon entropy. Under the conditional independence assumption, the entropy of the map is defined in (2).

1 $$\begin{aligned} p(g \mid z_{1:t}) = P(g = \textrm{occ} \mid z_{1:t}) \end{aligned}$$

2 $$\begin{aligned} H(G \mid z_{1:t}) = -\sum _{g \in G} \Big [ p(g \mid z_{1:t}) \log p(g \mid z_{1:t}) + (1 - p(g \mid z_{1:t})) \log (1 - p(g \mid z_{1:t})) \Big ]. \end{aligned}$$

#### Global NBV candidates selection

The search for the global NBV begins by extracting all frontiers, defined as the boundaries between known and unknown regions in the occupancy map. To reduce redundancy, the frontiers are clustered and downsampled using the Density-Based Spatial Clustering of Applications with Noise (DBSCAN) algorithm, yielding a set of representative candidate viewpoints. DBSCAN is applied with neighborhood radius $\epsilon _{db} = 0.5\,\textrm{m}$ and minimum cluster size set to 5. For each candidate point *v*, the potential information gain is evaluated across eight discrete yaw directions $\Theta = \{0^\circ ,45^\circ ,90^\circ ,135^\circ ,180^\circ ,225^\circ ,270^\circ ,315^\circ \}$. For each direction $\theta \in \Theta$, the set of observable cells $G^{\textrm{vis}}(v,\theta )$ is determined via ray-casting on the occupancy grid, constrained by the camera field of view $\phi = 90^\circ$ and maximum sensing range $R = 5.0\,\textrm{m}$. Only cells within the angular sector $[\theta -\phi /2,\theta +\phi /2]$ and distance *R* are considered, excluding those occluded by occupied cells.

The next step is to compute the information gain of each candidate viewpoint in all directions. The information gain *I* is defined as the reduction in map uncertainty when a new observation is made. The uncertainty of the map is measured using the entropy *H*(*G*), which is given in (2), where $z_{1:t}$ in $Z \subset \mathbb {R}$ represents the set of all observations up to time *t*. For a viewpoint *v* and direction $\theta$, the directional information gain is defined (3), where $Z(v,\theta )$ denotes the next observation obtained from viewpoint *v* facing direction $\theta$, and $G^{\textrm{vis}}(v,\theta )$ is the corresponding set of visible cells.

3 $$\begin{aligned} \begin{aligned} I(G; Z(v,\theta ) \mid z_{1:t})&= H(G \mid z_{1:t}) - H(G \mid Z(v,\theta ), z_{1:t}) = H(G^{\textrm{vis}}(v,\theta ) \mid z_{1:t}) \end{aligned} \end{aligned}$$

**Fig. 2:**
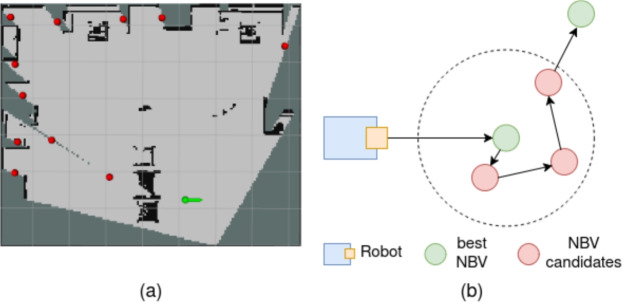
(**a**) is the result of finding global NBV. The red points are all NBV candidates, the green point is the selected NBV position, and the arrow is the NBV direction. (**b**) is the process of NBV selection between two global NBVs.

To incorporate motion effort, a cost factor *c* is introduced to account for both translational and rotational costs. This is particularly important under a limited camera field of view, where large yaw adjustments can significantly reduce exploration efficiency. The cost is defined in (4), where $d(x_t,v)$ denotes the path length from the current pose $x_t$ to viewpoint *v*, and $\Delta \theta (x_t,\theta )$ is the yaw change required to align the camera with direction $\theta$. The final utility score is computed in (5) and the global NBV is selected using the greedy rule, as in (6). The result of global NBV selection is illustrated in Fig. 2a. Algorithm 1 summarizes the process for calculating the global NBV.

4 $$\begin{aligned} c = \lambda _d d(x_t,v) + \lambda _\theta \Delta \theta (x_t,\theta ), \end{aligned}$$

5 $$\begin{aligned} \textrm{S}(v,\theta ) = \frac{I(v,\theta )}{c + \epsilon }, \end{aligned}$$

6 $$\begin{aligned} (v^{*},\theta ^{*}) = \arg \max _{(v,\theta )} \textrm{S}(v,\theta ). \end{aligned}$$

Due to the restricted FOV of a single camera, the robot may not fully observe the region around the selected global NBV $(v^*,\theta ^*)$ upon arrival. To address this limitation, a local completion phase is introduced. The set of local candidate viewpoints is defined in as $\mathscr {N}(v^*,\theta ^*) = \left\{ (v,\theta ) \,\Big |\, \Vert v-v^*\Vert \le \rho \right\}$, where $\rho$ is the local neighborhood radius. For each local candidate $(v,\theta ) \in \mathscr {N}(v^*,\theta ^*)$, the utility score (5) is evaluated, and viewpoints are iteratively selected using the greedy rule (6). This process continues until all candidates in $\mathscr {N}(v^*,\theta ^*)$ have been visited, ensuring sufficient coverage of the neighborhood around the global NBV even under a restricted camera FOV. The local completion process is illustrated in Fig. 2b.

**Algorithm 1:**
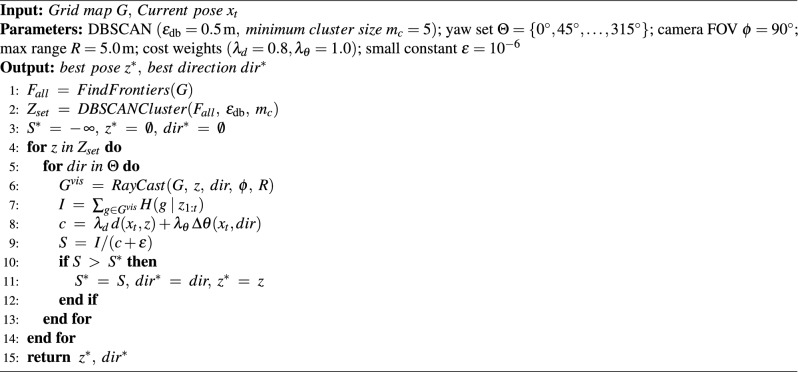
Find global NBV

### Dynamic object segmentation and tracking

To ensure robust localization in dynamic environments, the proposed SLAM system integrates semantic segmentation, multi-object tracking, and geometric consistency checks for dynamic object detection. Object segmentation is performed using YOLOv8s^36^, a state-of-the-art real-time detector trained on the COCO2017 dataset. This model can recognize and segment 80 object categories, including humans, vehicles, animals, and other commonly encountered objects. Consequently, the system is not limited to detecting pedestrians, but can also handle other mobile objects such as cars or movable chairs, which is important for general applicability in different environments. The choice of YOLOv8 is motivated by its high detection accuracy and real-time efficiency, which are critical for SLAM.

To maintain temporal consistency, BoT-SORT^37^ is applied as the multi-object tracker. BoT-SORT assigns consistent IDs to objects detected across consecutive frames, enabling the system to follow their trajectories over time. This provides essential information on object motion, which supports distinguishing static structures from moving entities and reduces the risk of introducing dynamic features into the map.

While semantic segmentation and tracking identify candidate dynamic objects, geometric verification based on the epipolar constraint is employed to confirm motion. During visual SLAM, features on static objects should satisfy the epipolar geometry:

7 $$\begin{aligned} \boldsymbol{P}_1 = [u_1, v_1, 1], \quad \boldsymbol{P}_2 = [u_2, v_2, 1], \end{aligned}$$

8 $$\begin{aligned} \boldsymbol{P}_2 \boldsymbol{F} \boldsymbol{P}_1^T = 0, \end{aligned}$$

where $\boldsymbol{P}_1$ and $\boldsymbol{P}_2$ are matched points in consecutive frames and $\boldsymbol{F}$ is the fundamental matrix. Feature correspondences are considered to consistently violate the epipolar constraint if their reprojection residual exceeds a predefined threshold for more than 3 consecutive frames, and these features are considered as dynamic features. By combining semantic cues with epipolar geometry, the system achieves robust dynamic object detection: semantic segmentation provides object-level priors, while epipolar consistency confirms whether those objects are indeed moving. Features associated with confirmed dynamic objects are removed from the SLAM optimization process, preventing drift and preserving map accuracy. In addition, the tracking of moving objects can also help with the NBV decision in local areas, which will be introduced in the next subsection.

### Local NBV decision

The local NBV decision is based on two parts: one part is the feature points distribution in the current frame, and the other part is the movement of the tracked moving objects. The local NBV module aims to decrease the influence of moving objects and make the robot move toward the direction that has a higher probability of features based on the generated FPM. The FPM is a combination of two probability maps, one coming from the extracted feature points, and one coming from the movement of dynamic objects.

#### Feature points

The probability map from static feature points is generated from the current camera frame. Suppose the current frame will be divided into *n* grids. For each grid, count the number of feature points. Then normalize the number of feature points in all regions so that the sum is one. The calculation of the probability map generated by feature points $\boldsymbol{M}_{f}$ is defined in (9). $N(G(\boldsymbol{x}))$ represents the number of feature points in the grid that the pixel in, $\boldsymbol{x} = [u, v]^T$ represents the pixel position, *K* is the total number of feature points. Finally, a Gaussian blur is introduced after constructing the normalized probabilistic map. Based on empirical found, a Gaussian blur applied to the normalized probabilistic map can smooth out local noise and grid boundary effects, providing a more continuous distribution that better reflects feature uncertainty and increases the stability of the FPM.

9 $$\begin{aligned} \boldsymbol{M}_{f}(\boldsymbol{x}) = \frac{N(G(\boldsymbol{x}))}{K}, \end{aligned}$$

This construction creates a discrete probability distribution over the image plane, representing the likelihood of observing static features at different locations. Then a Gaussian blur is introduced after constructing the normalized probabilistic map $\boldsymbol{M}_{f}$ to reduce local noise and grid boundary artifacts. Based on empirical found, a Gaussian blur applied to the normalized probabilistic map can smooth out local noise and grid boundary effects, providing a more continuous distribution that better reflects feature uncertainty and increases the stability of the FPM.

#### Tracked moving objects

To model the influence of dynamic objects, there are some transitions from tracked moving objects to the FPM. Dynamic objects reduce the likelihood of observing static features in regions they occupy or are expected to occupy. Since features inside dynamic objects are excluded from visual SLAM, the influence of dynamic objects is modeled by estimating the probability that a given pixel location may be affected by dynamic motion. So the problem can be transferred to the probability that dynamic objects may appear.

For each tracked dynamic object, pixels inside its segmentation mask are used to construct a probabilistic motion influence field. The probability that a pixel location $\boldsymbol{x}$ is affected by a dynamic object is modeled as a multivariate Gaussian distribution, which is shown in (10), where $\boldsymbol{\mu }$ denotes the pixel coordinate of a dynamic pixel inside the segmentation mask, and $\boldsymbol{\Sigma }_r$ is a rotated covariance matrix encoding motion uncertainty.

10 $$\begin{aligned} p(\boldsymbol{x}) = \frac{1}{2\pi \sqrt{|\boldsymbol{\Sigma }_r|}} \exp \!\left( -\frac{1}{2} (\boldsymbol{x}-\boldsymbol{\mu })^T \boldsymbol{\Sigma }_r^{-1} (\boldsymbol{x}-\boldsymbol{\mu }) \right) \end{aligned}$$

To account for directional motion, an anisotropic covariance matrix is constructed. For the anisotropic covariance matrix, the calculation is in (11) and (12). $\theta$ is the tracked object moving direction angle, $\boldsymbol{R}$ is the rotation matrix, $\boldsymbol{\Sigma }$ is the covariance matrix before rotation.

11 $$\begin{aligned} \boldsymbol{\Sigma } = \begin{bmatrix} \sigma _{\parallel }^2 & 0 \\ 0 & \sigma _{\perp }^2 \end{bmatrix}, \quad \boldsymbol{R}(\theta ) = \begin{bmatrix} \cos \theta & -\sin \theta \\ \sin \theta & \cos \theta \end{bmatrix} \end{aligned}$$

12 $$\begin{aligned} \boldsymbol{\Sigma }_r = \boldsymbol{R}(\theta )\boldsymbol{\Sigma }\boldsymbol{R}(\theta )^T \end{aligned}$$

The longitudinal and orthogonal variances $\sigma _{\parallel }$ and $\sigma _{\perp }$ can be calculated in (13), where *v* is the image-plane displacement of the tracked object centroid between consecutive frames (pixels per frame), $\epsilon$ is a small constant preventing degeneracy, and $\alpha$, $\beta$ are tunable parameters controlling uncertainty growth along and perpendicular to the motion direction. Tuning $\alpha$ and $\beta$ can adjust the level of the influence of speed on the uncertainty both along or perpendicular to the motion direction.

13 $$\begin{aligned} \sigma _{\parallel } = \alpha (v + \epsilon ), \qquad \sigma _{\perp } = \beta (v + \epsilon ) \end{aligned}$$

When multiple dynamic objects are present in the scene, each tracked object is modeled independently in the image domain. For each object, a Gaussian probability is generated over the pixels within its segmentation mask based on the predicted motion state. The dynamic influence map is obtained by summing the Gaussian probability of all tracked objects, capturing the cumulative reduction in feature observability caused by multiple movingh objects. Since segmentation masks of different objects do not overlap, each pixel is associated with a single dynamic influence value. The aggregated map is then normalized to produce a valid probability distribution in FPM fusion and construction the final FPM.

#### FPM fusion

The above equations calculated the probability that a pixel is dynamic based on one existing moving pixel. To construct the probability map $\boldsymbol{M}_t$, Gaussian kernels are placed at all *n* pixels inside the dynamic mask and added all together. The result is normalized over the entire image domain $\Omega$ to ensure it integrates to one. The final probability map generated from tracked moving objects can be calculated as in (14).

14 $$\begin{aligned} \boldsymbol{M}_{t}(\boldsymbol{x}) = 1 - \frac{\sum _{k=1}^{n} p_k(\boldsymbol{x})}{\sum _{\boldsymbol{x}\in \Omega }\sum _{k=1}^{n} p_k(\boldsymbol{x})}. \end{aligned}$$

Here, the subtraction from one represents that regions with higher dynamic probability should contribute less to feature probability. Finally, the FPM from moving objects $\boldsymbol{M}_t$ is linearly combined with the FPM from detected features $\boldsymbol{M}_f$ and normalized, as in (15).

Static feature observability and dynamic object influence are modeled as separable components in a pixel level and fused linearly prior to normalization to construct the final FPM. As a result, the final FPM $\boldsymbol{M}$ is obtained by linearly combining the probability maps both from features and dynamic objects and renormalizing, as shown in (15).

15 $$\begin{aligned} \boldsymbol{M}(\boldsymbol{x}) = \frac{\boldsymbol{M}_{f}(\boldsymbol{x}) + \boldsymbol{M}_{t}(\boldsymbol{x})}{\sum _{\boldsymbol{x}\in \Omega } \left( \boldsymbol{M}_{f}(\boldsymbol{x}) + \boldsymbol{M}_{t}(\boldsymbol{x})\right) }. \end{aligned}$$

**Fig. 3:**
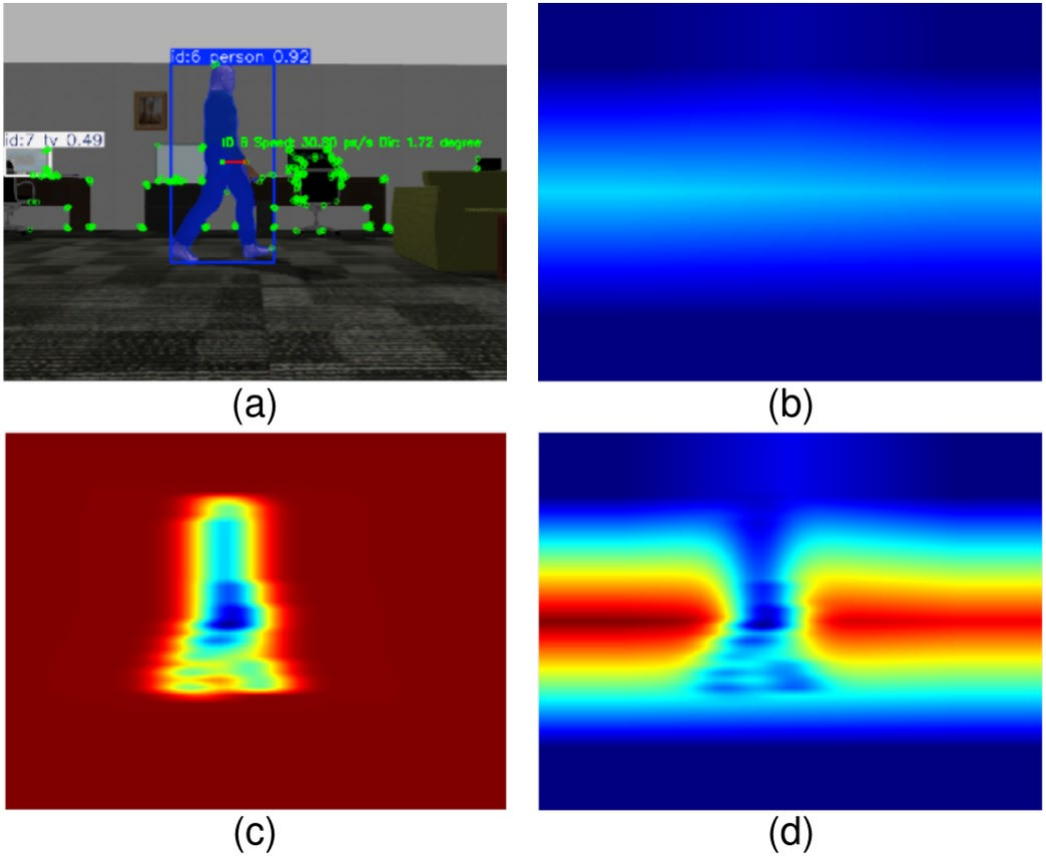
An example of FPM generation. (**a**) is the original frame with semantic segmentation, object tracking with KF, and feature detection (green points). (**b**) shows the probability map generated by feature points. (**c**) shows the probability map generated by the tracked moving object. (**d**) is the final FPM.

An example of the FPM construction process is shown in Fig. 3. Note that the probability map generated from dynamic objects represents diffused motion uncertainty rather than precise object contours.

**Fig. 4:**
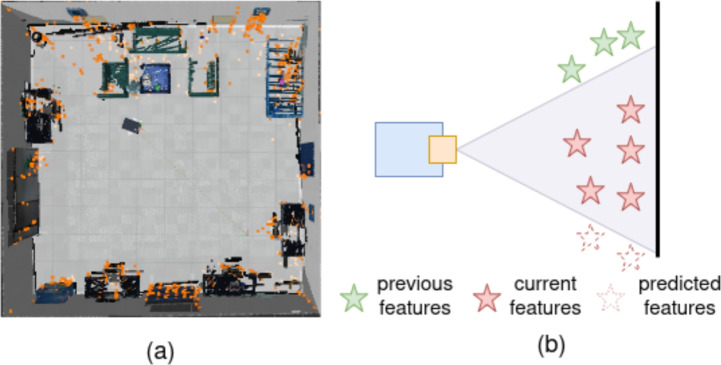
(**a**) shows the sparse feature points inside the map (points in orange color). (**b**) is the illustration of feature prediction. If the robot rotate up, the previous observed features and remaining current features will be the predicted features; if the robot rotate down to the unknown area, the predicted features will keep the same density.

With the FPM generated from the current state, estimating the information gain for a candidate’s next view requires predicting the FPM in the next predicted state. The predicted FPM is approximated using a deterministic prediction of the next state. This involves predicting both the feature distribution and the states of tracked moving objects. For the feature distribution, two cases are considered. If the candidate view includes previously unexplored regions, their feature density is approximated by that of the nearest adjacent explored area. If the view overlaps with an already mapped area, the feature count is obtained directly from the sparse map, which consists of reliable tracked feature points for robot localization. An example of the sparse feature map is shown in Fig. 4a, while the corresponding prediction process is illustrated in Fig. 4b. By getting feature density of the predicted view, the predicted FPM $\boldsymbol{M}_{f}^p(\boldsymbol{x})$ from the number of predicted features $N(G^p(\boldsymbol{x}))$ can be calculated using the same method in (9).

For tracked moving objects, the future state is predicted using a constant-velocity Kalman Filter (KF). Specifically, each pixel $\boldsymbol{x}$ inside the tracked object is associated with an independent KF instance, which updates position and velocity estimates and provides a prediction one step ahead under the assumption of constant speed. With the predicted pixel position $\boldsymbol{x}^p$, the predicted FPM from tracked objects $\boldsymbol{M}_{t}^p(\boldsymbol{x})$ can be calculated in (14).

With both the predicted feature distribution and predicted object states, the FPM for the next view $\boldsymbol{M}^p(\boldsymbol{x})$ is generated using (15). Suppose the observation for the next view is $Z^l$, the entropy of this predicted FPM $H(\boldsymbol{M}^p \mid Z^l,z_{1:t})$ can be calculated using (16). To find the local NBV, the information gain needs to be calculated for potential next views and find the maximum information gain. The information gain can be calculated in (17).

16 $$\begin{aligned} H(\boldsymbol{M}^p \mid Z^l, z_{1:t}) = -\sum _{m^p\in \boldsymbol{M}^p} p(m^p \mid Z^l, z_{1:t})\log p(m^p \mid Z^l, z_{1:t}), \end{aligned}$$

17 $$\begin{aligned} I(\boldsymbol{M};Z^l \mid z_{1:t}) = H(\boldsymbol{M} \mid z_{1:t}) - H(\boldsymbol{M}^p \mid Z^l, z_{1:t}). \end{aligned}$$

The robot is modeled as a differential-drive platform with velocity constraints on both linear and angular motion. Candidate poses are thus limited to those reachable within the dynamic window defined by these constraints, implemented using the Dynamic Window Approach (DWA)^39^. Local NBV planning is triggered at a fixed frequency (10 Hz in our experiments), ensuring that the robot continuously updates its motion plan as both features and tracked objects update. This combination of predicted FPM and dynamic feasibility ensures that the selected local NBV maximizes information gain while remaining executable under the robot’s kinematic limits. Algorithm 2 summarizes the process for calculating the local NBV.

**Algorithm 2:**
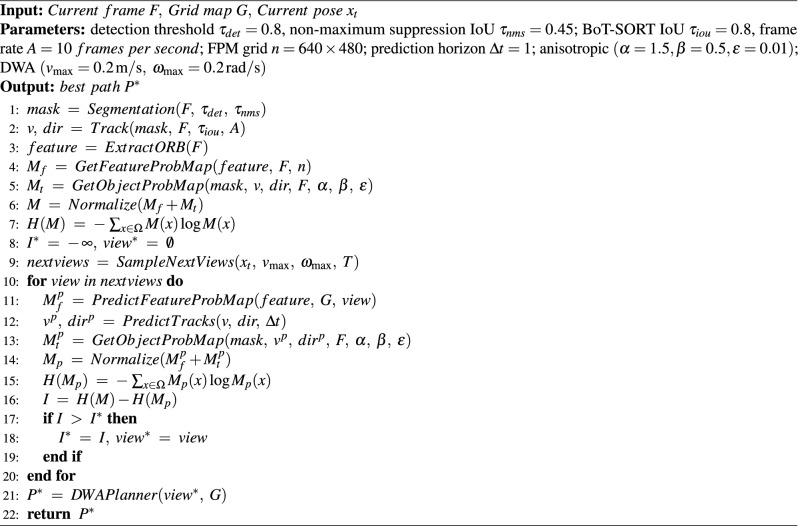
Find local NBV

## Experiments and results

For the experiments, the performance of the proposed SLAM system is evaluated both on two simulation environments and a real-world scene.

### Implementation details

Both simulation and real-world experiments are conducted using an RGB-D camera rigidly mounted on the robot platform. Camera intrinsics are obtained through offline calibration and remain fixed throughout all experiments. The calibrated intrinsic matrix coefficients $f_x$, $f_y$, $c_x$, $c_y$ are 573.4, 574.8, 320.1 and 322.6 respectively corresponding to a $640\times 480$ image resolution. Depth measurements are provided directly by the RGB-D sensor with a maximum reliable range of $5.0\,\textrm{m}$ and are registered to the RGB stream using factory calibration.

Semantic segmentation and object detection are performed using YOLOv8s model with pretrained weights trained on the COCO dataset with no extra finetuning. Detection confidence threshold is set to 0.8, and non-maximum suppression is performed with an IoU threshold of 0.45.

Detected semantic classes corresponding to potentially dynamic objects, only ’person’ is included as potential dynamic objects in experiments. Once a person is detected, the algorithm will identify whether it is moving using epipolar constraint as mentioned in 3.3.

Instance association across frames is handled by BoT-SORT with a maximum association age of 30 frames and an IoU matching threshold of 0.8. The resulting object tracks provide the motion in the pixel-level estimates used for dynamic uncertainty modeling.

### Experiments on simulation environments

**Fig. 5:**
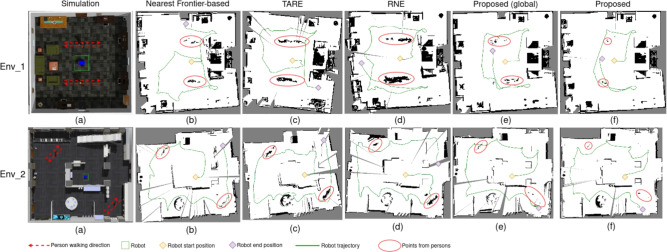
Active mapping in simulation environments.

The experiments are conducted on a computer that has an Intel Core i7-8700 CPU @ 3.20GHz $\times$ 12 processors and an NVIDIA GeForce RTX 3080 graphic. The operating system is Ubuntu 20.04. The experiments are conducted using the Robot Operating System (ROS)^40^, and the simulation environments are created in Gazebo^41^. The first environment has an open space with an area of 10 m $\times$ 10 m. The second environment has a more complex layout with a larger area of 19 m $\times$ 22 m. Both environments have two persons walking back and forth towards fixed trajectories. The simulation environments are shown in Fig. 5a Env_1 and Env_2. The linear and angular velocities of the robot are set to 0.2 m/s and 0.2 rad/s, grid map resolution is 0.05 m per cell. The robot will stop exploring after the next information gain from the global NBV module is less than a threshold. The threshold is 50 bits for Env_1 and 260 bits for Env_2.

**Fig. 6:**
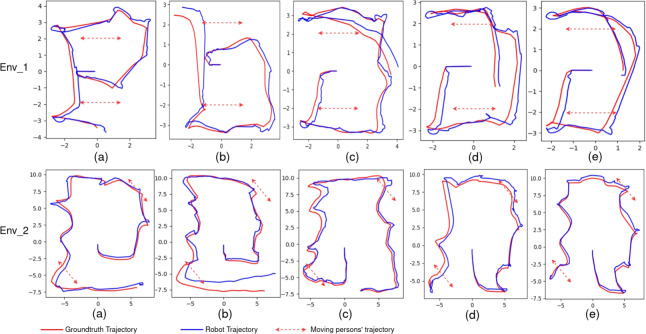
Comparison of groundtruth trajectories and robot trajectories. (**a**) Nearest Frontier based, (**b**) proposed (global only), (**c**) proposed.

The proposed method is divided into two parts: only the global NBV module part and the whole proposed method. They are compared with three state-of-the-art methods: the nearest frontier-based exploration method, RNEX^42^ and TARE^43^. Their efficiency is evaluated using travel distance, travel time, and map Entropy Reduction Rate (ERR). Their accuracy is evaluated using ATE, RPE. The active mapping results are shown in Fig. 5, and their travel distance, travel time, ATE, and ERR are shown in Tables 3 and 4. The robot trajectories and groundtruth trajectories are shown in Fig. 6.

The proposed approach is evaluated in two parts: using only the global NBV module and using the full hierarchical NBV framework. Both variants are compared with three representative exploration baselines, including a nearest-frontier strategy, RNEX^42^, and TARE^43^. Exploration efficiency is evaluated using travel distance, travel time, and the map Entropy Reduction Rate (ERR), which measures the rate of uncertainty reduction in the occupancy map. Localization accuracy is evaluated using standard SLAM metrics, including Absolute Trajectory Error (ATE), as well as Relative Pose Error (RPE) in both translational and rotational parts. They reflect the stability and the safety of motion execution in dynamic environments. Representative active mapping results are visualized in Fig. 5. More details of these metrics and the calculation methods are shown in Table 1 and Table 2. Quantitative comparisons of these metrics are shown in Table 3 and Table 4. The estimated robot trajectories and the corresponding ground-truth trajectories provided by the simulator are illustrated in Fig. 6.

**Table 1:** Definitions of evaluation metrics used in experiments.

Metric	Definition	Description
Travel Distance (m)	$\sum _{t=1}^{T-1}\Vert \textbf{p}_{t+1}-\textbf{p}_t\Vert$	Total path length of the robot
Travel Time (min)	$t_T - t_1$	Total exploration duration
ATE (m)	$\sqrt{\frac{1}{T}\sum _{t=1}^{T}\Vert \textbf{p}^{est}_t-\textbf{p}^{gt}_t\Vert ^2}$	Global localization accuracy
RPE Trans. (m)	$\sqrt{\frac{1}{N}\sum _{t}\Vert \Delta \textbf{t}^{est}_t-\Delta \textbf{t}^{gt}_t\Vert ^2}$	Local translational motion error
RPE Rot. (rad)	$\sqrt{\frac{1}{N}\sum _{t}\theta (\Delta \textbf{R}^{est}_t(\Delta \textbf{R}^{gt}_t)^{-1})^2}$	Local rotational motion error
ERR (bits/s)	$\frac{H(G_0)-H(G_T)}{T_{\textrm{travel}}}$	Information gain efficiency
Tracking loss rate (%)	$\frac{N_{\textrm{fail}}}{N_{\textrm{total}}}\times 100$	Frequency of tracking failures
Near collision counts	$N_{\textrm{nc}}$	Number of getting close to moving objects

**Table 2:** Notation used in evaluation metrics.

Symbol	Definition
$\textbf{p}_t$	Robot position at time step *t*
$\textbf{p}^{est}_t$	Estimated robot position at time *t*
$\textbf{p}^{gt}_t$	Ground-truth robot position provided by the Gazebo at time *t*
$\Delta \textbf{t}_t$	Relative translation between consecutive poses: $\textbf{p}_{t+1}-\textbf{p}_t$
$\Delta \textbf{R}_t$	Relative rotation between consecutive poses
$\theta (\cdot )$	Rotation angle extracted from a relative rotation matrix
*T*	Total number of poses in the trajectory
*N*	Number of valid relative pose pairs used for RPE computation
*H*(*G*)	Shannon entropy of the occupancy grid map *G*
$G_0,\,G_T$	Occupancy grid map at the start and end of exploration
$T_{\textrm{travel}}$	Total exploration time in seconds
$N_{\textrm{fail}}$	Number of SLAM tracking failures
$N_{\textrm{total}}$	Total number of evaluated frames
$N_{\textrm{nc}}$	Number of near collision times detected in simulation

The results of the experiment show that the proposed method outperforms other methods both in efficiency and accuracy. As shown in Fig. 5, particularly in Fig. 5e and f, the method incorporating the local NBV module leaves the fewest residual points corresponding to moving persons (highlighted in red circles) on the map, indicating that dynamic targets have minimal impact on the mapping process. From Table 3 and  4, compared to the nearest frontier-based method, the global NBV module takes less time because it can maximize the entropy reduction, making it complete the mapping process more efficiently. Compared with TARE and RNE, maps generated by TARE exhibit greater distortion and yield the highest ATE and RPE due to interference from dynamic objects. Compared to only the global NBV module, the whole proposed method has the least ATE, RPE and travel time because it avoids the person in advance to make its path smoother and leads itself to the next area with rich features once it detects the moving person, which increases the accuracy.

Regarding localization accuracy, Fig. 6 compares the estimated and groundtruth trajectories. The nearest-frontier, TARE, and RNE methods exhibit higher drift when the robot approaches moving obstacles. In contrast, the proposed method demonstrates the smallest trajectory drift because of the local NBV module, which reduces the likelihood of keeping moving persons within the robot’s current FOV and thus improves localization precision.

In Env_2, the proposed method also has the least ATE. However, regarding the efficiency, the global NBV module is slightly better than the whole proposed method. This is because in this experiment, when the robot was navigating using the whole proposed method, the robot deviated from its original path based on the local NBV module the first time it encountered the moving person, and later navigated back to the original global goal. This will compromise a little efficiency for a higher localization accuracy because of less dynamic influence.

To isolate the contribution of the local NBV and FPM module to system robustness and safety, tracking loss rate and near collision counts are evaluated for the two proposed variants: the only global NBV configuration and the full hierarchical NBV framework. This comparison is conducted within the same SLAM, detection, and tracking pipeline, the only difference between the two variants is whether the local NBV module is activated to refine viewpoints in the neighborhood of a global goal using the FPM. Tracking loss is directly decided by the SLAM backend when localization fails during the exploration. Near collision counts are counted when the robot remains within a distance of 2 meters from moving obstacles for more than 3 seconds, which represent a safety factor during exploration. The results are shown in Table 5.

The experiments show that the full hierarchical NBV framework leads to a reduction in both tracking loss rate and near collision counts across the two environments. In Env 1, the tracking loss rate decreases from $30\%$ under the only global variant to $10\%$ with the full hierarchical NBV, while near collision counts are reduced from 5 to 1. Env 2 shows the same trend with tracking loss rate reduced from $20\%$ to 10 and near collision counts reduced from 7 to 2. These results indicate that the local NBV and FPM module provides big robustness benefits beyond global frontier selection alone. By performing local viewpoint completion under limited FOV constraints, the robot is able to maintain more stable feature observations and reduce interactions with moving obstacles.

**Table 3:** Comparison results of environment 1. The best results are highlighted.

Method	Nearest frontier	TARE	RNE	Proposed (global)	Proposed
Travel distance (m)	31.25	25.31	34.34	29.88	24.21
Travel time (min)	5.24	4.89	4.93	4.91	4.31
ATE (m)	0.30	0.32	0.31	0.27	0.25
RPE Trans. (m)	0.27	0.28	0.29	0.25	0.21
RPE Rot. (rad)	0.15	0.18	0.17	0.19	0.13
ERR (bits/s)	77.35	85.90	85.27	84.43	97.47

**Table 4:** Comparison Results of environment 2. The best results are highlighted.

Method	Nearest frontier	TARE	RNE	Proposed (global)	Proposed
Travel distance (m)	71.79	69.07	71.06	**68.04**	68.86
Travel time (min)	11.44	11.32	11.79	**10.54**	10.60
ATE (m)	0.42	0.56	0.46	0.38	**0.30**
RPE Trans. (m)	0.32	0.44	0.39	0.31	**0.26**
RPE Rot. (rad)	0.21	0.22	0.21	0.23	**0.18**
ERR (bits/s)	152.97	153.88	148.71	**166.94**	165.36

**Table 5:** Comparison of tracking loss rate and numbers of getting near collision between global NBV and full hierarchical NBV in two environments.

	Environment 1		Environment 2	
	Proposed (global)	Proposed	Proposed (global)	Proposed
Tracking loss rate (%)	30	10	20	10
Near collision counts	5	1	7	2

### Experiments on a real-world scene

#### Experiment environment

**Fig. 7:**
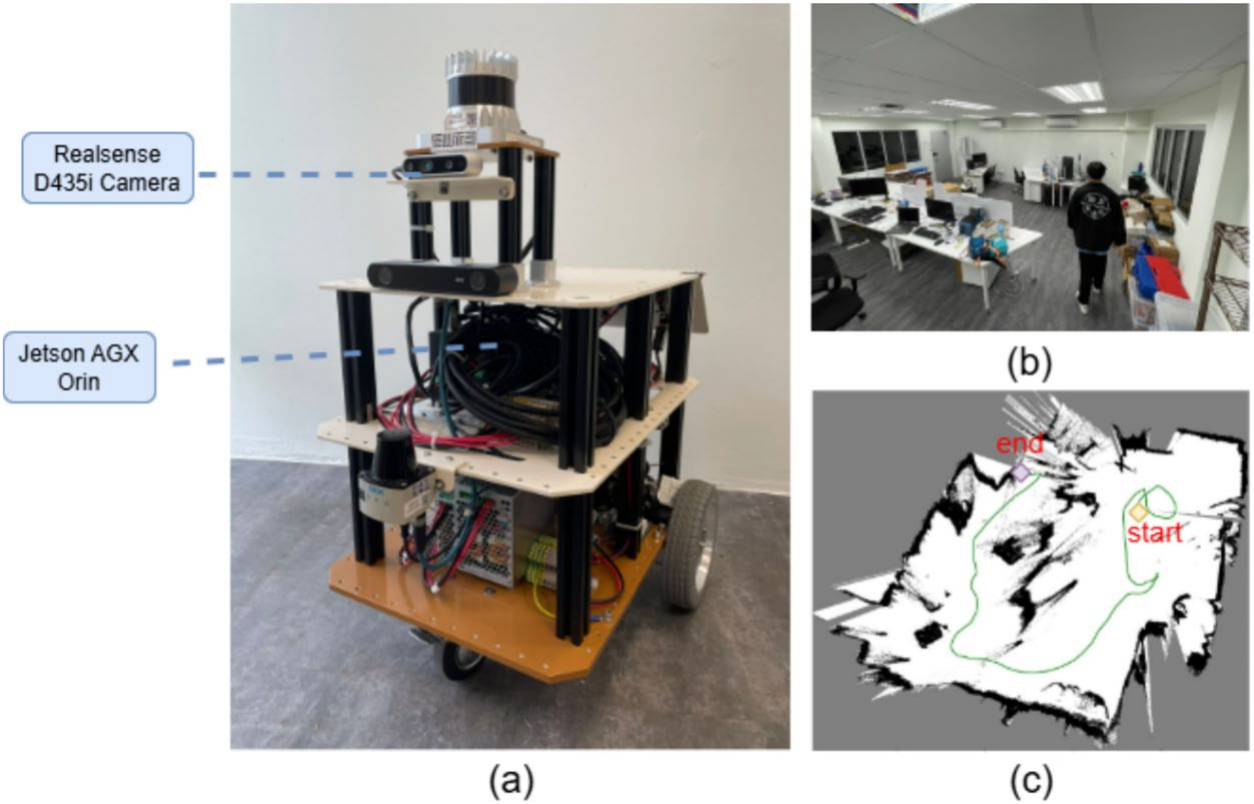
The real-world experiment. (**a**) is the Meerkat robot; (**b**) is the experiment environment, a person is walking back and forth; (**c**) is the created map with the robot trajectory.

The real-world experiment was conducted using the robot Meerkat^44^. It is equipped with a Jetson AGX Orin, which has an NVIDIA Ampere architecture GPU and an Arm Cortex-A78AE CPU. The camera is Realsense D435i. The robot and experiment environment are shown in Fig. 7a and b, the map created by the robot is shown in Fig. 7c. The real-world experiment validates the usability of the proposed method in real applications.

#### Computational complexity and real-time analysis

The proposed framework has four main components: global NBV planning, local NBV planning based on the FPM, semantic segmentation and object tracking, and SLAM back-end optimization. The global NBV module operates on a 2D occupancy grid with $N_g$ cells and $N_v$ frontier candidates. For each candidate viewpoint, information gain is evaluated over a fixed set of discrete yaw angles, resulting in a computational complexity of $\mathscr {O}(N_v \cdot N_g^{\textrm{vis}})$, where $N_g^{\textrm{vis}} \ll N_g$ denotes the number of visible cells obtained via ray casting. Since the global NBV module is triggered at a low frequency, its impact on online performance is negligible. The local NBV module works in the image domain. Let $N_p$ denote the number of pixels in the image and $N_d$ the number of dynamic object pixels. FPM construction scales linearly with the number of detected features and image grids, which is $\mathscr {O}(N_p)$. Dynamic object uncertainty modeling using anisotropic Gaussian diffusion also scales as $\mathscr {O}(N_d)$. For each candidate local view, the predicted FPM and corresponding entropy are computed, generating a total complexity of $\mathscr {O}(N_c \cdot N_p)$, where $N_c$ is the number of locally reachable candidate views. Overall, the proposed framework has linear complexity related to map size and image resolution. The performance of real application is shown in Table 6.

**Table 6:** Real-time performance of each system module on the Meerkat robot.

Module	Time (s)	Hardware
SLAM pipeline	0.132	CPU
Semantic segmentation (YOLOv8)	0.032	GPU
Object tracking (BoT-SORT)	0.024	GPU
Global NBV planning	0.087	CPU
Local NBV planning	0.064	CPU
Motion planning	0.056	CPU

Table 6 shows the average runtime of each system module measured during real-world experiments on the Meerkat robot. Semantic segmentation and object tracking continuously update dynamic object information on the GPU, while the CPU updates the SLAM algorithm, the whole global and local NBV modules, and robot motion planning. These results demonstrate that the proposed hierarchical NBV framework can be deployed on practical robotic platforms in real time.

## Conclusion

This paper introduces a novel active SLAM system based on Shannon mutual information, designed to achieve robust localization and efficient mapping in dynamic environments. The proposed system uses semantic segmentation to exclude moving objects, determines the global NBV using an occupancy map, tracks moving objects, and identifies the local NBV based on the current feature distribution and the status of moving objects. Experimental results from two simulations demonstrate the system’s ability to maintain robustness and provide accurate localization in dynamic environments. Additionally, real-world experiments show that the system is suitable for real-time robotic applications. While this method offers new insights into active SLAM in dynamic settings, some limitations remain. The robot may still lose track when dynamic objects suddenly occupy the majority of the camera view. Future work will focus on two areas: enabling the robot to recover from sudden tracking loss and integrating a new path planning module to enhance mapping efficiency.

## Supplementary Information

Supplementary Information.

## Data Availability

All data supporting the findings of this study are available within the paper and its Supplementary Information.
